# Hypercholesterolemia-Induced HDL Dysfunction Can Be Reversed: The Impact of Diet and Statin Treatment in a Preclinical Animal Model

**DOI:** 10.3390/ijms23158596

**Published:** 2022-08-02

**Authors:** Leonie Schoch, Pablo Sutelman, Rosa Suades, Laura Casani, Teresa Padro, Lina Badimon, Gemma Vilahur

**Affiliations:** 1 Cardiovascular Program ICCC, Institut de Recerca, Hospital Santa Creu i Sant Pau, IIB Sant Pau, 08025 Barcelona, Spain; lschoch@santpau.cat (L.S.) psutelman@gmail.com (P.S.); rsuades@santpau.cat (R.S.); lcasani@santpau.cat (L.C.); tpadro@santpau.cat (T.P.); lbadimon@santpau.cat (L.B.); 2 Faculty of Medicine, University of Barcelona (UB), 08036 Barcelona, Spain; 3 CiberCV, 08025 Barcelona, Spain; 4 Cardiovascular Research Chair, Autonomous University of Barcelona (UAB), 08025 Barcelona, Spain

**Keywords:** HDL dysfunction, hypercholesterolemia, diet, statin, pig

## Abstract

High-density lipoproteins (HDL) undergo adverse remodeling and loss of function in the presence of comorbidities. We assessed the potential of lipid-lowering approaches (diet and rosuvastatin) to rescue hypercholesterolemia-induced HDL dysfunction. Hypercholesterolemia was induced in 32 pigs for 10 days. Then, they randomly received one of the 30-day interventions: (I) hypercholesterolemic (HC) diet; (II) HC diet + rosuvastatin; (III) normocholesterolemic (NC) diet; (IV) NC diet + rosuvastatin. We determined cholesterol efflux capacity (CEC), antioxidant potential, HDL particle number, HDL apolipoprotein content, LDL oxidation, and lipid levels. Hypercholesterolemia time-dependently impaired HDL function (−62% CEC, −11% antioxidant index (AOI); *p* < 0.01), increased HDL particles numbers 2.8-fold (*p* < 0.0001), reduced HDL-bound APOM (−23%; *p* < 0.0001), and increased LDL oxidation 1.7-fold (*p* < 0.0001). These parameters remained unchanged in animals on HC diet alone up to day 40, while AOI deteriorated up to day 25 (−30%). The switch to NC diet reversed HDL dysfunction, restored apolipoprotein M content and particle numbers, and normalized cholesterol levels at day 40. Rosuvastatin improved HDL, AOI, and apolipoprotein M content. Apolipoprotein A-I and apolipoprotein C-III remained unchanged. Lowering LDL-C levels with a low-fat diet rescues HDL CEC and antioxidant potential, while the addition of rosuvastatin enhances HDL antioxidant capacity in a pig model of hypercholesterolemia. Both strategies restore HDL-bound apolipoprotein M content.

## 1. Introduction

Meta-analyses of interventions aimed at raising high-density lipoprotein (HDL) cholesterol (HDL-C) [1] and Mendelian randomization studies [2] have challenged the accepted hypothesis that a decrease in cardiovascular (CV) risk can be achieved by increasing HDL-C levels. In fact, recent population studies have associated abnormally high concentrations of HDL-C with increased CV risk [3,4]. Altogether, these data argue against the use of plasma HDL-C concentration as a reliable indicator of HDL CV protective functions and shift the attention towards assessing HDL functional activity rather than HDL-C content. In fact, the measurement of HDL function has shown a superior ability to predict CV risk compared to quantitative measurements of HDL-C levels [5].

HDLs are complex particles composed of multiple proteins, lipids, and miRNAs that can exert multiple functions [6,7,8]. As such, beyond their well-known ability to limit atherosclerosis progression by enhancing cholesterol removal from vascular foam cells to the liver for excretion (i.e., reverse cholesterol transport), HDL particles have shown to exert antioxidant, anti-inflammatory, and anti-thrombotic properties, to promote endothelial protection, and limit myocardial ischemia/reperfusion injury [6,7]. However, an increasing number of studies suggest a loss or attenuation of HDL protective functions in the presence of coronary artery disease (CAD) and comorbidities including inflammation [9], diabetes mellitus [10], metabolic syndrome [11], and chronic kidney disease [12]. Furthermore, we recently demonstrated in different preclinical animal models that high plasma levels of low-density lipoprotein cholesterol (LDL-C) suffice to impair the ability of HDL particles to regress atherosclerotic plaques [13] and limit cardiac damage in the setting of myocardial infarction [14]. We also identified changes in HDL structural components (proteins, lipids, and miRNAs) that may partly explain the functional loss of HDL CV benefits [15,16]. Based on these previous findings, we now seek to determine whether hypercholesterolemia-induced HDL dysfunction can be reversed. There are hints towards improvements in HDL function after LDL apheresis in patients with familial hypercholesterolemia, indicating a causal role for circulating cholesterol [17,18]. For this purpose, we examine, in a preclinical pig model of diet-induced hypercholesterolemia, in which hypercholesterolemia is based on intestinal absorption with compensatory abrogation of hepatic cholesterol synthesis, whether the implementation of standard of care lipid-lowering approaches (diet and rosuvastatin) reverses hypercholesterolemia-induced changes in HDL particles.

## 2. Results

### 2.1. Dynamics of Hypercholesterolemia-Induced HDL Dysfunction

**Figure 1:**
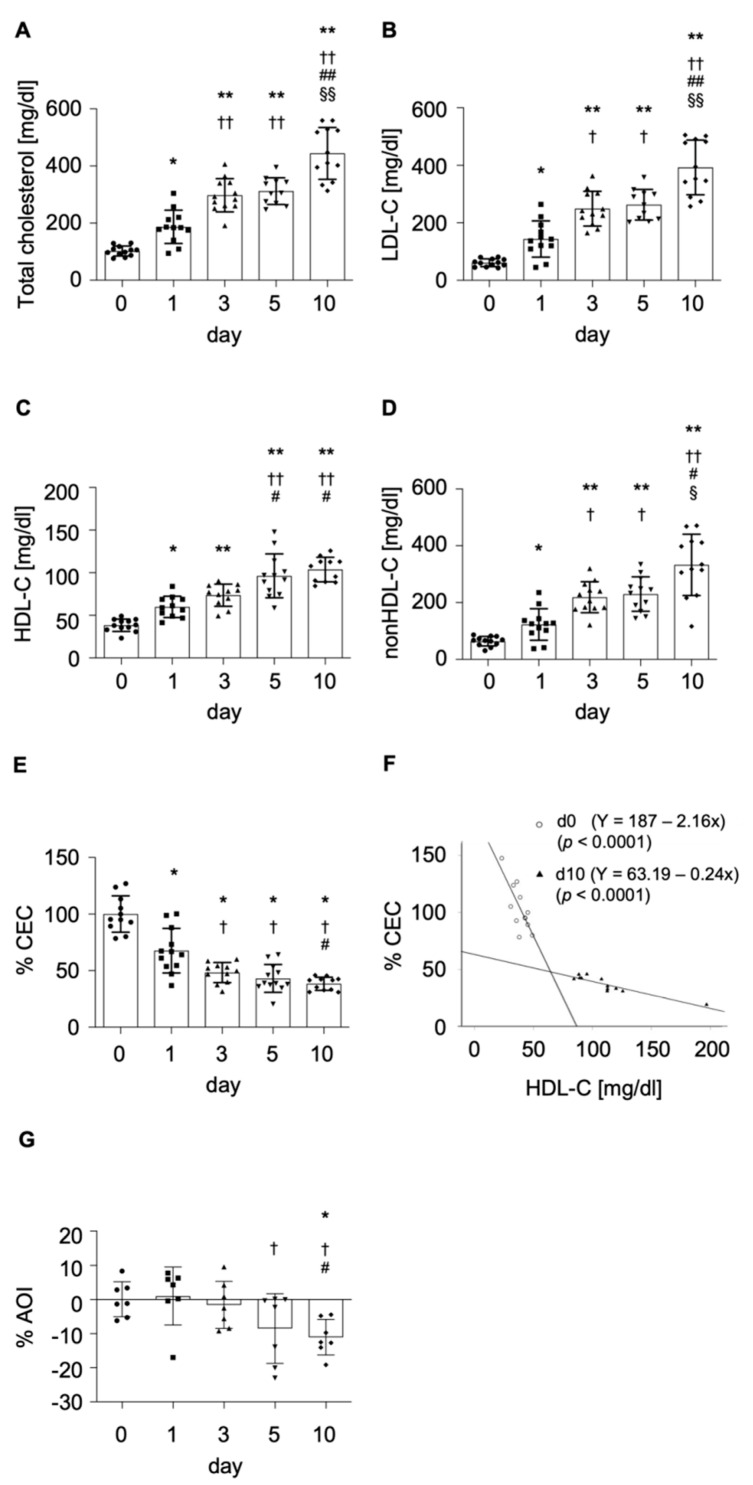
Diet-induced hypercholesterolemia onset increases lipid parameters while HDL function is impaired. Progression of plasma lipid parameters including total cholesterol (**A**), LDL-C (**B**), HDL-C (**C**), non-HDL-C (**D**), and HDL functional measures cholesterol efflux capacity (CEC) from macrophages to HDL (**E**), and antioxidant capacity (**G**) are depicted during the first 10 days of HC diet. The correlation between cholesterol efflux capacity and HDL-C levels (d0 and d10 (**F**) is shown with linear regressions. Individual values are depicted with bars representing the mean ± SD over time. Shapiro–Wilk test confirmed normality (alpha = 0.05), and data were analyzed by one-way ANOVA with Tukey’s multicomparison test and considered significant with a *p*-value < 0.05. * *p* < 0.05, ** *p* < 0.0001 in comparison to day 0; † *p* < 0.05, †† *p* < 0.0001 in comparison to day 1; # *p* < 0.05, ## *p* < 0.0001 in comparison to day 3; §§ *p* < 0.0001, § *p* < 0.05 in comparison to day 5. LDL-C: low-density lipoprotein cholesterol; HDL-C: high-density lipoprotein cholesterol; CEC: cholesterol efflux capacity; AOI: antioxidant index.

Diet-induced hypercholesterolemia was confirmed over the 10-day period by a steady and significant time-dependent increase in total cholesterol (TC), LDL-C, HDL-C, and non-HDL-C levels (Figure 1A–D). Intake of HC diet acutely impaired CEC by 32% at day 1, reaching a plateau at day 3 (further 19% reduction; *p* = 0.0056; Figure 1E). There was a strong negative correlation between CEC and HDL-C levels (slope: −0.008 vs. slope: −0.001; Figure 1F). Regarding HDL antioxidant capacity, this was found to be impaired at day 5 with an 11% reduction compared to baseline (*p* = 0.0070; Figure 1G), and it progressively worsened up to day 25 (see below).

### 2.2. Impact of Diet and Rosuvastatin on HDL Function

Once we characterized the dynamics of hypercholesterolemia-induced HDL dysfunction, we addressed the impact of NC diet or rosuvastatin intervention on restoring HDL function.

#### 2.2.1. HDL Cholesterol Efflux Capacity

**Figure 2:**
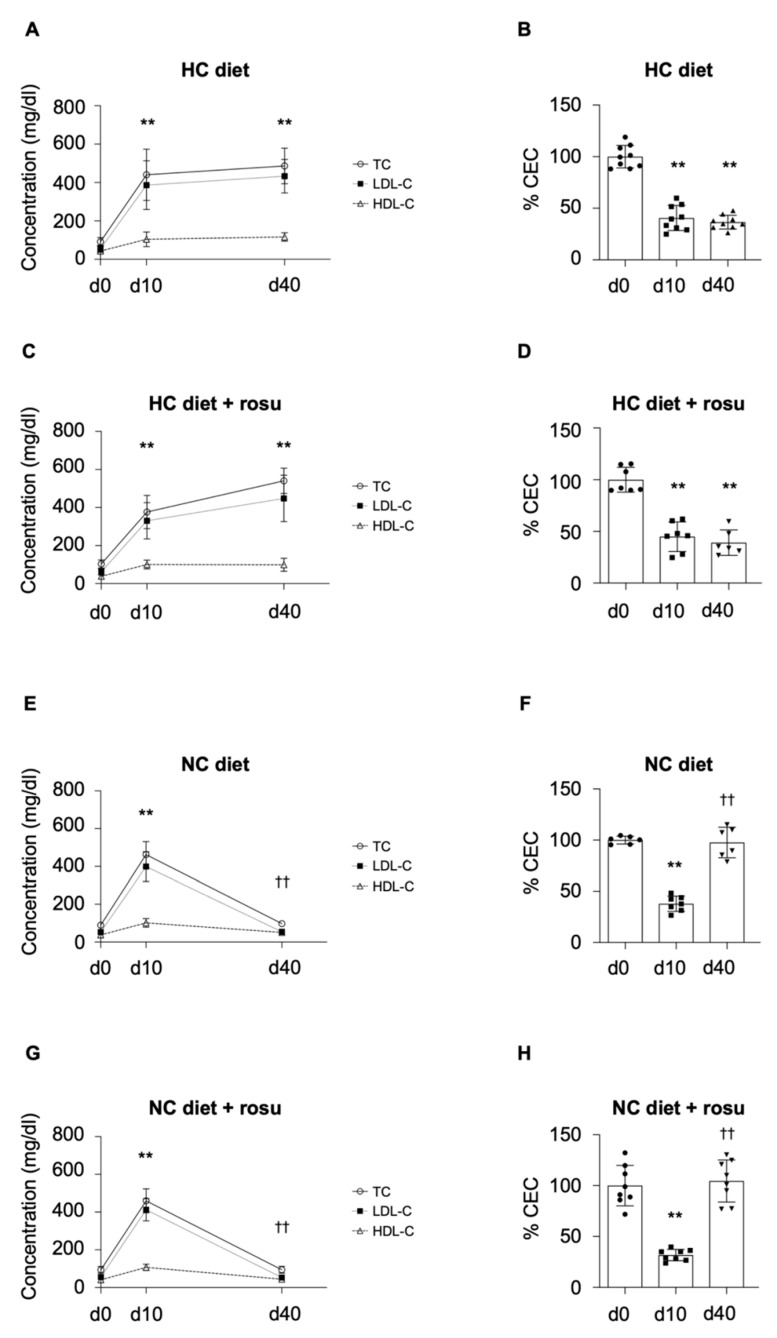
Effects of diet and rosuvastatin intervention on lipid parameters and CEC. Changes in plasma total cholesterol (TC, circle with continuous line), low-density lipoprotein cholesterol (LDL-C, square with dotted line), and high-density lipoprotein cholesterol (HDL-C, triangles with dashed line) levels (**A**,**C**,**E**,**G**), and percent cholesterol efflux from macrophages to HDL (% Efflux; **B**,**D**,**F**,**H**) are depicted as mean (bars or line through respective symbol) ± SD (whiskers) for all four groups over the period of 40 days. Shapiro–Wilk test confirmed normality (alpha = 0.05), and data were analyzed by unpaired t-test and considered significant with a *p*-value < 0.05. ** *p* < 0.0001 in comparison to day 0; †† *p* < 0.0001 in comparison to day 10. CEC: cholesterol efflux capacity; TC: total cholesterol; HDL: high-density lipoprotein cholesterol; LDL: low-density lipoprotein cholesterol; HC: hypercholesterolemic; NC: normocholesterolemic; rosu: rosuvastatin; d0–40: day 0–40.

Animals that remained on HC diet up to day 40 showed no further increase in cholesterol levels (TC, LDL-C, and HDL-C; Figure 2A) nor further impairment in CEC (Figure 2B), as compared to day 10. Administration of rosuvastatin on top of HC diet did not induce any changes in lipid concentrations nor HDL CEC, as compared to those animals fed HC diet alone (Figure 2A–D), in agreement with the cholesterol-absorption model without hepatic synthesis. In contrast, animals that switched to NC diet reduced their lipid levels (*p* < 0.0001; Figure 2E) and improved CEC (*p* < 0.0001; Figure 2F) to levels comparable to those observed at baseline (day 0; physiological state). Administration of rosuvastatin on top of NC diet did not result in a superior efficacy to reduce cholesterol levels (Figure 2G) or enhance CEC (Figure 2H), as compared to animals fed NC diet alone. We also evaluated the impact of diet and rosuvastatin intervention on lipid levels and CEC relative to their HC state at day 10. The relative difference (Δ) between HC baseline (day 10) and study endpoint (day 40) as per TC, LDL-C, HDL-C, and CEC was significantly different between both diets but not within the same diet with and without rosuvastatin for TC, LDL-C, HDL-C, and CEC (Supplementary Figure S1A–D). In summary, adding rosuvastatin to either of the diets did not exert any effect on cholesterol levels and CEC.

As observed for up to day 10, cholesterol levels (TC, LDL-C, HDL-C) were inversely correlated with CEC (*p* < 0.0001; Supplementary Figure S2). Moreover, a clear clustering per day was observed between functional and healthy HDL particles on day 0 and functionally impaired HDLs on day 10 (Figure 1F).

#### 2.2.2. HDL Antioxidant Index

**Figure 3:**
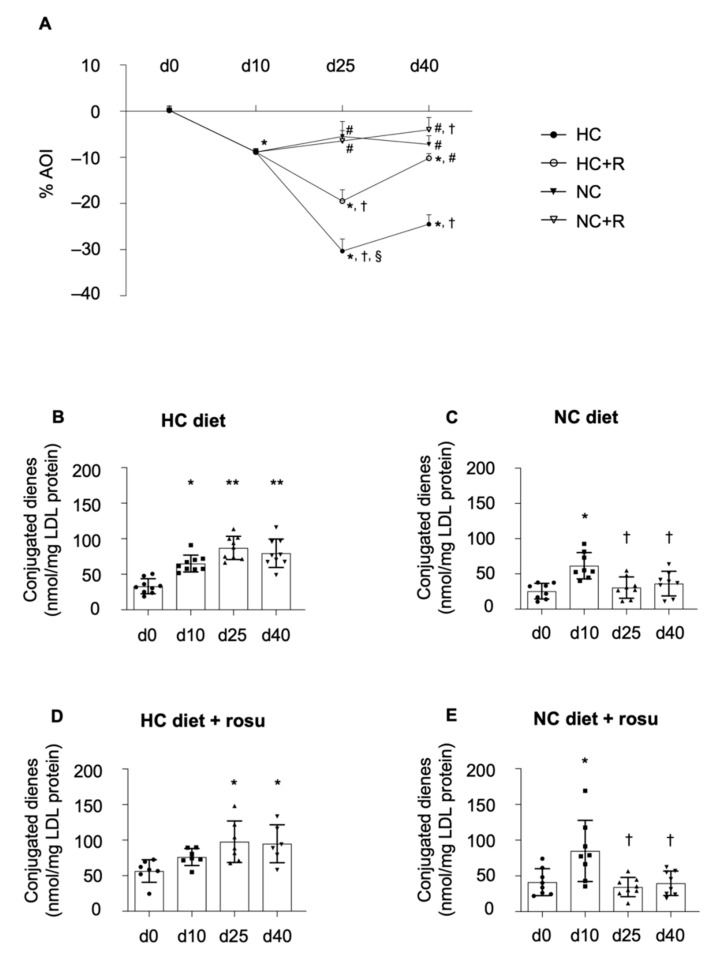
Effects of diet and rosuvastatin intervention on HDL antioxidant capacity. Changes in HDL antioxidant capacity are presented as percent antioxidant index ((**A**), % AOI; measure for capacity to reverse LDL oxidation) and levels of conjugated dienes ((**B**–**E**) product of lipid oxidation). Data are shown as mean ± SEM ((**A**); for visual clarity of the graph) or SD (whiskers) over the period of 40 days. (**A**) includes all four groups, whereas (**B**–**E**) represent each group individually. Shapiro–Wilk test confirmed normality (alpha = 0.05), and data were analyzed by one-way ANOVA with Tukey’s multicomparison test and considered significant with a *p*-value < 0.05. For (**A**): * *p* < 0.05 in comparison to day 0; † *p* < 0.05 in comparison to day 10; # *p* < 0.05 compared to HC; § *p* < 0.05 in comparison to NC. For (**B**–**E**): * *p* < 0.05, ** *p* < 0.0001 in comparison to day 0; † *p* < 0.05 in comparison to day 10. d0–40: day 0–40; HC: hypercholesterolemic diet; NC: normocholesterolemic diet; +R/+rosu: + rosuvastatin; LDL: low-density lipoprotein.

Administration of HC diet up to day 40 further impaired HDL AOI by 25%, as compared to day 10, reaching a plateau at day 25 (Figure 3A). Administration of rosuvastatin on top of the HC diet prevented hypercholesterolemia-induced deterioration of HDL AOI at day 40 (Figure 3A). The switch to NC diet alone did not exert any improvement on HDL AOI, as compared to day 10, whereas administration of rosuvastatin on top of NC diet more effectively improved HDL AOI at study endpoint (Figure 3A; *p* < 0.05 vs. day 10). The relative difference between the AOI at HC baseline (day 10) and study endpoint (day 40) was significantly increased in NC diet ± rosuvastatin in comparison to HC diet alone (*p* < 0.0001; Supplementary Figure S1E). Of note, rosuvastatin treatment on top of HC diet significantly improved ΔAOI in comparison to HC diet alone (*p* < 0.0001), and rosuvastatin on top of a NC diet showed an improvement as compared to HC + rosuvastatin.

#### 2.2.3. Formation of Conjugated Dienes

The formation of conjugated dienes was significantly increased in animals on HC diet, reaching a plateau at day 10 that persisted up to day 40 (Figure 3B; *p* < 0.0001). The switch to NC diet showed a rapid decline in the formation of conjugated dienes at day 25 to levels comparable to those found at baseline (Figure 3C; *p* = 0.0029). The addition of rosuvastatin to either of the diets did not alter conjugated dienes formation, as compared to either diet alone (day 10–40; Figure 3B–E).

The relative difference of conjugated diene levels (ΔCD) between day 10 and study endpoint (day 40) was significantly decreased in animals fed NC diet with rosuvastatin in comparison to HC diet ± rosuvastatin (*p* < 0.05; Supplementary Figure S1F).

### 2.3. Liver HMG-CoA Reductase Activity

Rosuvastatin-administered pigs showed a significant 23% reduction in liver HMG-CoA reductase activity, as compared to non-treated animals (Supplementary Figure S3; *p* = 0.0096).

### 2.4. Impact of Diet and Rosuvastatin on HDL Particle Number

Ten days of HC diet caused a significant 2.8-fold increase in total HDL-P (*p* < 0.0001; Table 1). HDL-P remained unchanged in animals maintained on HC diet, whereas animals that switched to NC diet displayed a marked reduction in HDL-P to levels comparable to baseline (Table 1; *p* < 0.0001). The addition of rosuvastatin to either of the diets exerted no effect on total HDL-P count (Table 1).

The relative difference of HDL-P between day 10 and study endpoint differed between diets but not with the addition of rosuvastatin to either of the diets (*p* < 0.0001; Supplementary Figure S1G).

### 2.5. Impact of Diet and Rosuvastatin on HDL Apolipoprotein Content

**Figure 4:**
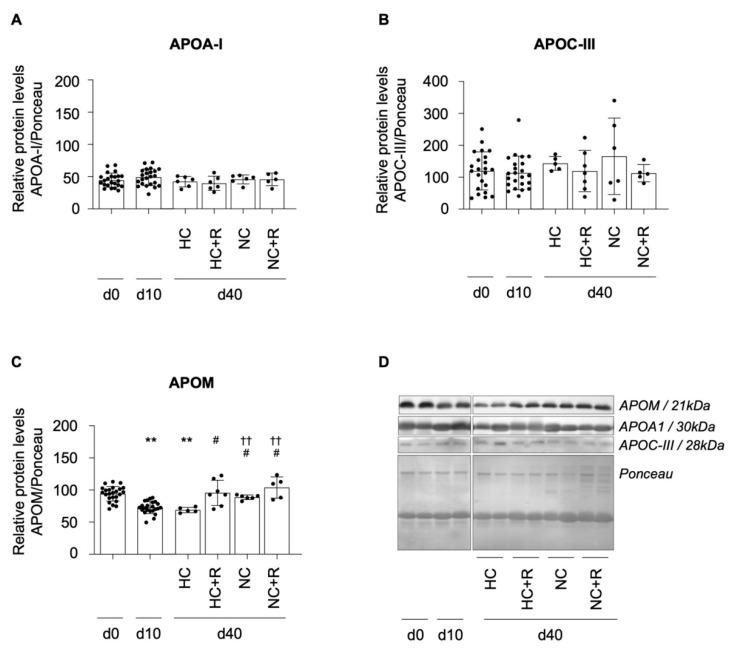
Effects of diet and rosuvastatin intervention on HDL apolipoprotein levels. Changes in protein levels of apolipoproteins (APO) APOA-I (**A**), APOC-III (**B**), and APOM (**C**) in isolated HDL particles are presented as relative quantification and representative Western blots (**D**) at study endpoint. Western blot quantifications are shown as mean ± SD. Shapiro–Wilk test confirmed normality (alpha = 0.05), and data were analyzed by unpaired t-test and considered significant with a *p*-value < 0.05. ** *p* < 0.0001 in comparison to day 0; †† *p* < 0.0001 in comparison to day 10; # *p* < 0.05 in comparison to day 40 of animals on HC diet. d0–40: day 0–40; HC: hypercholesterolemic diet; NC: normocholesterolemic diet; +R: + rosuvastatin.

We finally evaluated the impact of diet and rosuvastatin on the presence of main apolipoproteins (APO) transported by HDL, including APOA-I, APOM, and APOC-III. HDL-bound APOA-I (Figure 4A,D) and APOC-III (Figure 4B,D) levels did not change over time and among all animal groups. In contrast, HDL-bound APOM content was reduced by 22% after a 10-day HC diet regime (Figure 4C; *p* < 0.0001), in line with our previous findings [15]. Yet, we further evidenced that such reduction remained up to day 40 (*p* < 0.0001) in animals maintained on HC alone. In contrast, addition of rosuvastatin to the HC diet or switching to NC diet significantly restored HDL APOM content to levels found at baseline (Figure 4C; *p* < 0.009). Of note, addition of rosuvastatin treatment to NC-diet-fed animals showed a marked trend towards a further increase in HDL APOM levels in comparison to NC diet alone (Figure 4C; *p* = 0.0556).

The relative difference of HDL-bound APOM levels between day 10 and day 40 showed a significant increase in both rosuvastatin-treated conditions, as compared to HC diet alone (*p* < 0.05; Figure 1H).

### 2.6. Animal Follow-Up

No differences were observed for glucose levels or liver and kidney parameters among all groups and throughout the study (Supplementary Table S1).

## 3. Discussion

Growing evidence within the last years indicates that pathological conditions render HDL particles dysfunctional [6]. We demonstrated that high plasma levels of LDL-C impair HDL-related cardiovascular protective functions [13,14,15]. We now prove that in the setting of diet-induced hypercholesterolemia, a model experiencing high intestinal cholesterol absorption and little hepatic cholesterol synthesis, there is a significant increase in HDL-P, but the particles show a dysfunctional behavior. The dynamics of HDL dysfunction are, however, reversible. As such, lowering LDL-C levels by implementing a low-fat diet rescues HDL CEC and antioxidant potential after a 30-day intervention, while the addition of rosuvastatin more effectively enhances HDL antioxidant capacity. Furthermore, we also prove that both strategies restore the content of the HDL-bound key cardioprotective lipoprotein APOM.

In line with our previous findings, we detect a significant increase in plasma lipid levels after a 10-day ingestion of HC diet, whereas no changes are observed after chronic ingestion of regular chow [14,15,19,20]. Yet, we further demonstrate that, although lipid parameters show a time-dependent increase during the 10-day onset of diet-induced hypercholesterolemia, HDL CEC is already impaired at day 1 post HC diet initiation, reaching a plateau at day 3. So far, rapid HDL adverse remodeling and subsequently reduced CEC has been described in response to acute inflammation [6]. This change is thought to be orchestrated by a wide variety of secreted cytokines and proteins roughly peaking at 24–48 h post-stimulus [6]. Herein, we evidence, for the first time, of an acute (24 h) and progressive (up to day 10) deleterious effect of LDL-C levels on HDL CEC. HDL antioxidant capacity was also found to be affected in the short term (5 days), yet it progressively worsened, reaching a plateau at day 25. These data evidence that HDL functional properties have differing sensitivity to elevated LDL-C levels. As such, HDL CEC is found to be acutely affected by a partial increase in LDL-C plasma concentrations, whereas HDL AOI is altered upon a persistent exposure to maximal LDL-C concentrations.

We further explored the potential ability of dietary and statin intervention to rescue HDL function. So far, only a few studies have investigated the impact of nutraceuticals (PUFAs, MUFAs, antioxidant supplements) on restoring a particular HDL function [21]. Yet, these studies only addressed certain HDL-associated changes, such as CEC, paraoxonase 1 mass or activity, and the oxidative status of the particle assessed by the presence of oxidative metabolites. We provide a comprehensive study and time course analyses on the benefits of lowering LDL-C by dietary change and statin treatment on overall HDL function and apolipoprotein content with the aim to ascertain the impact of conventional therapeutic approaches addressed to manage hypercholesterolemia [22]. As such, we assessed individual and potential synergistic effects of dietary cholesterol reduction and statin treatment on HDL function (CEC and antioxidant potential) and apolipoprotein composition. We firstly demonstrate that the normalization of cholesterol levels by switching from HC to NC diet is associated with a complete restoration of HDL CEC. On the one hand, we observe no changes in cholesterol levels by the addition of rosuvastatin to the diet. While animal models in rabbits, rats, and mice have mainly shown reduced cholesterol levels upon statin treatment [23], pig models might differ in regard to the time of statin treatment needed to achieve significant reductions of cholesterol levels. Busnelli et al. reported significant reductions in total as well as LDL cholesterol after 70 days on high-dose atorvastatin (80 mg/d) in pigs on high-fat diet (5% cholesterol) [24], and Matthan et al. showed that 6 months of lower atorvastatin doses (20–40 mg/d) were needed to significantly reduce LDL-C levels in pigs on high-fat diet (1.5% cholesterol) [25]. Furthermore, Li et al. detected no changes in total cholesterol levels in high-fat-diet-fed LDL-R knockout pigs after a 4-month pitavastatin treatment [26], and Boodhwani et al. reported reduced total cholesterol levels in hypercholesterolemic Yucatan miniswines after a 5-month atorvastatin treatment (not after 1 month, as in our study) at comparable statin doses [27]. Yet, the detected significant reduction in HMG-CoA reductase activity serves as proof of oral intake of the drug and supports that longer treatment periods are required to achieve a decline in cholesterol levels.

As per the impact of statins on HDL CEC, some studies evidenced benefits after 6–10 weeks of statin treatment [28,29,30], whereas others did not [31,32]. Although we observed no changes in cholesterol levels within our studied timeframe, the potential mechanisms by which longer statin treatment periods may interfere with HDL cholesterol removal remain to be investigated, mostly considering no changes in APOA-I content, as discussed below.

Interestingly, we further detect a strong negative correlation between HDL-C levels and CEC. We had already evidenced, in the same animal model, that hypercholesterolemia induced larger and dysfunctional HDL particles [15] which, in concurrence with higher HDL particle numbers detected herein, supports the need to assess HDL function rather than HDL-C levels. Preliminary findings in a case control study of individuals with extremely high HDL-C and CAD also support the same claim [33].

As per the impact of diet on HDL antioxidant potential, although serum cholesterol levels reached a plateau on day 10, AOI continued to deteriorate up to day 25, indicating a negative impact of high and persistent LDL-C plasma levels on HDL function. Although a switch to NC diet prevented further impairment, it did not completely restore HDL function. Interestingly, however, rosuvastatin administration enhanced HDL antioxidant potential. Besides their well-described lipid-lowering properties after longer treatment periods, statins are known to exert antioxidant properties [34]. In fact, most studies have demonstrated statin-related systemic antioxidant effects [35]. As such, statins have been reported to dose-dependently increase the expression or activity of antioxidant enzymes/factors, including superoxide dismutase (SOD) [36], catalase (CAT) [37], glutathione (GSH) [38], glutathione s-transferase (GST) [37], and glutathione peroxidase (GPX) [38]. Our data suggest that a 30-day rosuvastatin treatment is able to modify HDL antioxidant capabilities (AOI), an effect not observed for LDL. In this latter regard, a previous study in hypercholesterolemic patients showed that a 12-week statin treatment is required to reduce the formation of conjugated dienes in LDL [39]. Altogether, these findings indicate a different susceptibility of HDL and LDL particles to statin treatment. Indeed, longer follow-up studies are required to provide further insights into the impact of statins on LDLs.

We and others report that HDL dysfunction is closely associated with the occurrence of protein-related structural changes [15]. We detected no changes in HDL APOA-I content in any animal group, excluding any potential effect of both diet and rosuvastatin on the amount of this apolipoprotein chiefly involved in macrophage cholesterol removal [15]. We also found that HDL APOC-III content was not affected by diet or statin treatment throughout our experimental study. A previous study reported the ability of atorvastatin to affect plasma levels of APOC-III [40]. As such, 5 weeks of 40 mg/daily reduced plasma APOC-III in metabolic syndrome patients. In contrast, different dietary interventions (e.g., MUFA, high-fat, low-fat) have been shown to exert no effects on APOC-III levels [41,42]. Yet, only one previous study in a cohort of mixed individuals with and without CAD or diabetes mellitus investigated HDL-bound APOC-III, reporting no significant changes upon statin treatment [43].

In contrast, we detect changes in the amount of APOM transported by HDLs. We firstly observe a marked reduction in the APOM content in the presence of hypercholesterolemia [15] and further evidence that both diet and rosuvastatin treatment are able to restore HDL-bound APOM levels to baseline. Simvastatin has previously been shown to increase hepatic transcriptional and protein levels of APOM [44], likely through liver nuclear transcription factors (e.g., PPAR) [45,46], although the mechanisms by which this occurs remain to be fully determined. APOM is the main carrier for the cardioprotective signaling molecule sphingosine-1-phosphate and is negatively associated with CV risk [47]. Furthermore, Vaidya et al. recently showed that S1P drives cholesterol efflux via the ABCA1/ApoA-I axis, emphasizing the contribution of both S1P and ABCA/ApoA-I axis in macrophage cholesterol removal [48]. Since we do not detect changes in ApoA-I levels, we could speculate either that longer rosuvastatin treatment periods are required to induce potential changes in this apolipoprotein or that hypercholesterolemia, per se, may induce alterations in the main cholesterol efflux receptors, ABCA1 (ABCG1 and SR-B1), that are not reverted after a 30-day rosuvastatin treatment. Longer treatment periods may be required to modify the components mainly involved in cholesterol removal.

## 4. Materials and Methods

The study protocol and procedures were approved by the Institutional Animal Care and Use Committee and authorized by the Animal Experimental Committee of the local government (No. 9340) in accordance with the Spanish law (RD 53/2013) and European Directive 2010/63/EU. All procedures conform to the Guide for the Care and Use of Laboratory Animals published by the US NIH (No. 85-23, revised 1985), follow the ARRIVE guidelines 2.0 [49], and are committed to the 3Rs of laboratory animal research.

### 4.1. Experimental Design

**Figure 5:**
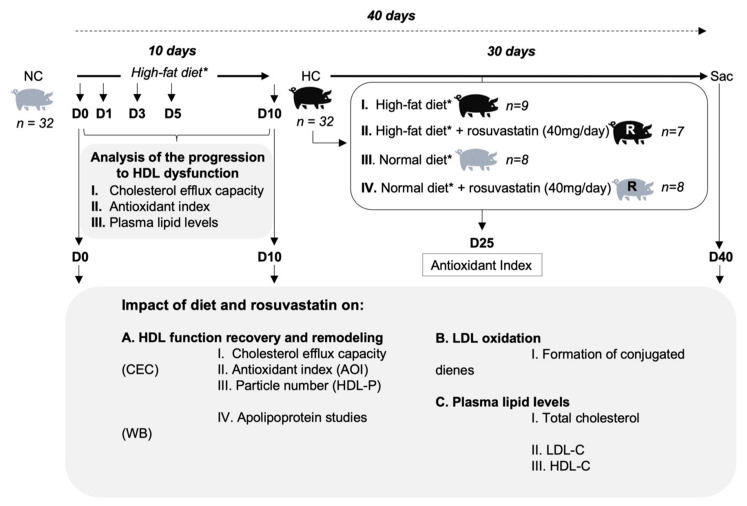
Study design. All animals were fed a hypercholesterolemic diet for the first 10 days of the study. From this day onwards, the animals were randomly divided into four groups to receive 30 days of (I) hypercholesterolemic diet alone (n = 9), (II) hypercholesterolemic diet with 40 mg rosuvastatin daily (n = 7), (III) normocholesterolemic diet alone (n = 8), (IV) normocholesterolemic diet with 40 mg rosuvastatin daily (n = 8). Samples were collected at indicated timepoints. HDL: high-density lipoprotein; CEC: cholesterol efflux capacity; AOI: antioxidant index; WB: Western blot. * High-fat and normal diet refer to hypercholesterolemic and normocholesterolemic diet, respectively.

The study design is shown in Figure 5. Four-month-old female Duroc–Landrace crossbred pigs (n = 32; weight: 33.1 ± 5.3 kg) were fed a Western-type hypercholesterolemic (HC) diet for 10 days. From day 10 up to day 40 (total of 30 days), animals were randomly distributed into the following four arms: (I) were kept on HC diet (n = 9), (II) were fed HC diet with 40 mg/daily rosuvastatin (n = 7), (III) switched to a regular normocholesterolemic (NC) diet (n = 8), and (IV) switched to NC diet with 40 mg/daily rosuvastatin (n = 8). Rosuvastatin was administered orally once daily before feeding time.

The two diets were composed of the following ingredients, expressed as percent of total energy (Table 2):

We previously demonstrated that a 10-day intake of this HC diet renders HDL particles dysfunctional and results in a significant increase in cholesterol plasma levels, which is comparable to those found in hypercholesterolemic patients [15]. Yet, the dynamics of HDL loss of function and its association with lipid levels remain unknown. Hence, first of all, we performed a substudy in order to characterize HDL dysfunction over this 10-day period in a subgroup of 12 randomly selected animals. For this purpose, 20 mL of blood was drawn from the femoral artery after 8 h of fasting at day 0 (baseline), days 1, 3, and 5 (hypercholesterolemia induction), and day 10 (established hypercholesterolemia) for the assessment of cholesterol levels, HDL cholesterol efflux capacity (CEC), and HDL antioxidant index (AOI). Thereafter, we examined the impact of diet and rosuvastatin on HDL function and remodeling over a 30-day period (up to day 40). For this purpose, blood was drawn at baseline, day 10 (established hypercholesterolemia), day 25 (halfway through treatment), and day 40 (study endpoint), for the assessment of HDL AOI, HDL CEC, HDL particle number (HDL-P), and HDL apolipoprotein content. We also assessed the susceptibility of LDL particles to oxidation and plasma lipid levels. At study endpoint (day 40), the liver was collected, and the activity of the statin target 3-hydroxy-3-methyl-glytaryl-coenzyme A (HMG-CoA) reductase was determined.

### 4.2. Lipoprotein Particle Isolation

HDL (density range of 1.063–1.210 g/mL) and LDL (density range of 1.019–1.063 g/mL) particles were isolated from serum by sequential ultracentrifugation as described before [50]. Briefly, the serum was adjusted to a density of 1.019 g/mL by a concentrated potassium bromide salt solution and centrifuged at 225,000× *g* (18 h) in a Beckman L-60 ultracentrifuge with a fixed angle type 50.4 Ti rotor (Beckman, Brea, CA, USA). After removing the top layer containing very low- and intermediate-density lipoproteins (VLDL and IDL), the density of the infranatant was adjusted to 1.063 g/mL, centrifuged (225,000× *g*, 20 h), and LDL particles were collected from the top fraction. The process was repeated, adjusting serum density to 1.210 g/mL and centrifuging at 225,000× *g* (24 h, 4 °C). Floating HDL particles were collected from the top fraction.

Isolated LDL and HDL particles were dialyzed against 1X phosphate buffered saline (1X PBS) for 24 h, and total protein content was quantified by a colorimetric assay based on bicinchoninic acid (BCA assay; Pierce, Thermo Fisher Scientific, Waltham, MA, USA). Samples were either kept well protected from light at 4 °C, for functional studies, or frozen at −20 °C, for protein and NMR analyses. We already standardized this method to ensure ApoB-free, microvesicle-free, and exosome-free HDL particle preparations [13,15]. A pool of control LDLs for assessing HDL antioxidant capacity was isolated from sera obtained from NC control pigs and was processed as described above.

### 4.3. Assessment of HDL Functionality

#### 4.3.1. HDL Cholesterol Efflux Capacity

CEC was determined at days 0, 10, and 40 using cholesterol-laden murine macrophages as previously described [51]. Briefly, J774A.1 mouse macrophages were cultured in RPMI 1640 medium (11530586 by Gibco, Thermo Fisher) containing 10% of heat-inactivated FBS, 2 mM glutamine, 100 U/mL penicillin, 100 U/mL streptomycin, and 10 ug/mL gentamycin at 37 °C in a humidified atmosphere of 5% CO_2_. Cells (seeding density of 1.5 × 10^5^ cells per well in a 6-well culture dish) were labeled with [1α,2α (n)-^3^H] cholesterol (GE Healthcare, Chicago, IL, USA) at 1 μCi per well for 48 h. The labeled cells were equilibrated in 0.2% BSA overnight and thereafter incubated with RPMI media containing 5% ApoB-depleted serum (4 h at 37 °C) to induce cholesterol efflux from the [^3^H]-labeled cells. Radioactivity was measured by liquid scintillation counting both in the supernatant and the cells, and the percentage of cholesterol efflux was calculated by expressing the radioactive cholesterol released to the medium as a fraction (%) of the total radioactive cholesterol present in the well (sum of radioactive cholesterol in cells and supernatant). The final data were normalized to HDL particle number, and baseline values were set to 100%. We also normalized the data to HDL-C, which led to the same fold change (data not shown).

#### 4.3.2. HDL Antioxidant Index

We assessed HDL AOI at days 0, 10, 25, and 40. HDL AOI is based on the capacity of HDL particles to actively reverse LDL oxidation using 2,7-dichlorofluoresceindiacetate (H2DCFDA) (Invitrogen Inc., Carlsbad, CA, USA) and copper-oxidized LDL particles (Kalen Biomedical Inc., Savage, MD, USA). The assay was performed as previously described [52]. Briefly, HDL and pooled control LDLs were isolated from pig serum by sequential ultracentrifugation, as described above. The LDL pool (250 μg/mL in 1X PBS) was oxidized in the presence of 0.5 mM copper sulfate for 4 h at 37 °C. LDL oxidation was stopped by adding 25 μL of 5 mM EDTA stock solution. Oxidized LDL (25 μL of a 250 μg/mL solution) alone and a mixture of oxidized LDL plus 6.4 μL of individual HDL samples (100 μg/mL in 1X PBS) were placed in a round-bottom, black polypropylene microtiter plate (Corning, Sigma Aldrich, Sant Louis, MO, USA) and incubated at 37 °C (24 rpm, 1 h). DCFH-DA was dissolved in fresh methanol at 2.0 mg/mL and incubated at room temperature protected from light for 30 min to release DCFH. The DCFH solution was diluted 10-fold, and 25 μL of this working solution was added to each well (including the wells without lipoproteins), mixed, and incubated in the dark at 37 °C (24 rpm 2 h). The interaction with lipid oxidation products converts DCFH to DCF, which produces intense fluorescence. Fluorescence intensity was determined using a fluorescent microplate reader (Infinite 200 PRO, Tecan, Männedorf, Switzerland) set at an excitation wavelength of 485 nm and an emission wavelength of 530 nm. Results are presented as the antioxidant capacity of HDL particles, which indicates the potential of HDL to actively counteract LDL oxidation (percent AOI). Data were normalized by the baseline mean of AOI.

#### 4.3.3. Accumulation of Conjugated Dienes in LDL Particles (Lipid Oxidation)

We assessed the formation of conjugated dienes (susceptibility of lipid oxidation) as an indirect measure of HDL antioxidant capacity. LDL particles were freshly isolated from individual pig serum by sequential ultracentrifugation, as described above. Conjugated dienes have an absorbance peak at 234 nm and were measured on a SpectraMax 190 Microplate Reader (Molecular Devices, Philadelphia, PA, USA) in UV-STAR microplates (Greiner Bio-One, Frickenhausen, Germany) in 100 µg/mL LDL particles of each animal. The data are represented as nanomole of conjugated dienes per mg of LDL protein.

### 4.4. HDL Particle Number by NMR

HDL particles were analyzed by nuclear magnetic resonance (NMR) in 500 µL plasma using diffusion coefficients to provide a direct measure of the mean particle numbers, as previously described [53]. Briefly, particle concentration and diffusion coefficients were obtained from the measured amplitudes and attenuation of their spectroscopically distinct lipid methyl group NMR signals using the 2D diffusion-ordered 1H NMR spectroscopy (DSTE) pulse.

### 4.5. HDL Content of Apolipoproteins

Total protein concentrations of isolated HDL particles from all animals were determined by the BCA Assay (Pierce, Thermo Fisher Scientific, Waltham, MA, USA). Equal amounts of protein per sample were loaded onto SDS-PAGE gels, and, following protein separation, proteins were transferred to nitrocellulose membranes (wet transfer, 400 mA, 2 h). Membranes were blocked for 1 h at room temperature with either 5% BSA or 5% milk in 1X TBS-T (0.05% Tween 20) and incubated with the respective primary antibodies (overnight, 4 °C). We assessed protein levels of the HDL apolipoproteins APOA-I (ab231718 by Abcam), APOM (MBS2001617 by MyBioSource), and APOC-III (710262 by Invitrogen) based on our previous findings [15]. Volume intensity of the bands was determined with the ChemiDoc™ XRS system (BioRad Hercules, CA, USA) in chemiluminescence detection mode and Quantity-One 4.6.8 software. Data are represented as relative protein levels normalized to Ponceau staining.

### 4.6. HMG-CoA Reductase Activity

Liver HMG-CoA reductase activity was assessed in liver samples of HC and NC animals with (n = 8) and without (n = 8) rosuvastatin treatment (40 mg/daily) obtained at study endpoint (day 40). Tissue samples from livers of all animals were isolated, snap-frozen, homogenized to tissue powder, and resuspended in ice-cold 1X PBS (0.25 g/mL) containing protease inhibitors. The samples were thoroughly resuspended and sonicated in eight 30-second intervals. The lysate was centrifuged at 1500× *g* and 4 °C for 10 min and total protein concentration of the supernatant was determined by BCA Assay (Pierce, Thermo Fisher Scientific, Waltham, MA, USA). Liver HMG-CoA reductase activity was measured by a commercially available colorimetric enzyme activity assay (CS1090 Sigma-Aldrich, St. Louis, MO, USA), as indicated in the manufacturer’s instructions, in 1.2 µg of total protein lysates. Results are depicted as percent HMG-CoA reductase activity, and non-treated values were set to 100%.

### 4.7. Follow-Up of Biochemical and Hematological Parameters

For follow-up measurements, 20 mL of blood was drawn from the femoral artery of fasting animals (8 h) for all time points. Lipid and glucose levels, as well as liver and renal parameters, were assessed throughout the study (Figure 1 and Supplementary Table S1). Triglycerides and total, as well as HDL, cholesterol levels were measured in plasma by a semiautomated biochemical analyzer (CLIMA MC15, RAL S.A., Spain), and LDL-C was calculated by the following formula:

$$\left[\mathrm{LDL}-C\right]=\left[\mathrm{total}\;\mathrm{cholesterol}\right]-\left[\mathrm{HDL}-C\right]-\frac{\left[\mathrm{triglycerides}\right]}{5}$$

Glucose, creatinine, total protein, and GOT were determined in serum by dedicated kits from RAL S.A., Spain.

### 4.8. Statistics

Statistical analyses were performed using GraphPad Prism 8.1.1 (San Diego, CA, USA) and SPSS 25.0 software (IBM SPSS Statistics, New York, NY, USA). Data are presented as mean ± standard deviation (SD), and individual values are depicted where indicated. *p*-values < 0.05 are considered statistically significant. The Shapiro–Wilk test was applied to determine normal distribution of the data, and intergroup comparisons were performed by two-sided, unpaired Student’s t-test. Grouped analyses were performed by nonparametric Kruskal–Wallis with Dunn’s multicomparison test or parametric one-way ANOVA/mixed-effects analysis with Tukey’s multicomparison test. Correlations between lipid parameters and HDL CEC were assessed by Spearman correlation and linear regression.

## 5. Conclusions

Higher HDL function (particularly CEC) is associated with lower adverse cardiovascular outcomes [54,55,56], underlining the need to identify strategies capable of restoring HDL key protective effects. In the present study, we demonstrate that lowering dietary fat intake with the consequent drop in cholesterol plasma levels acutely and persistently restores HDL function. Moreover, although rosuvastatin exerts no pleiotropic effects on HDL CEC, it does enhance HDL antioxidant properties and also restores HDL-APOM content. Of note, no changes in liver and kidney parameters were observed in those animals administered rosuvastatin, allowing us to exclude any short-term detrimental effects of the treatment (Supplementary Table S1).

## Figures and Tables

**Table 1:** Effects of diet and rosuvastatin intervention on HDL particle number. Data are reported as mean ± SD. HC: hypercholesterolemia; NC: normocholesterolemia; R: rosuvastatin.

Sample	HDL-P (µmol/L)	*p*-Value
(a) **Day 0**	17.9 ± 2.7	<0.0001 (vs. b,c,d)
(b) **Day 10**	50.2 ± 13.6	<0.0001 (vs. a,e,f)
**Day 40**		
(c) HC	54.0 ± 9.5	<0.0001 (vs. a,e,f)
(d) HC + R	56.4 ± 15.0	<0.0001 (vs. a,e,f)
(e) NC	18.5 ± 2.6	<0.0001 (vs. b,c,d)
(f) NC + R	16.8 ± 3.2	<0.0001 (vs. b,c,d)

**Table 2:** Dietary composition of hypercholesterolemic and normocholesterolemic diet.

Component	HC Diet (SSniff)	NC Diet (Sanky)
Energy	4881 kcal/kg	4019 kcal/kg
Total fat	24%	4%
of which is saturated fat	21%	
of which is cholesterol	2%	
of which is cholic acid	1%	
Proteins	20%	20%
Carbohydrates	35%	53%
Fiber	5%	6%
Minerals	6%	8%
Water	10%	9%

## Data Availability

The data that support the findings of this study are available from the corresponding author upon reasonable request.

## Supplementary Materials

The supporting information can be downloaded at https://www.mdpi.com/article/10.3390/ijms23158596/s1.
