# Supplementary material for: Hypercholesterolemia-Induced HDL Dysfunction Can Be Reversed: The Impact of Diet and Statin Treatment in a Preclinical Animal Model
Source: Int J Mol Sci. 2022 Aug 2;23(15):8596. doi: 10.3390/ijms23158596 (PMC9368958; doi:10.3390/ijms23158596)
Supplement: Supplementary file 1 [file ijms-23-08596-s001.zip › ijms-1797991-supplementary.pdf]

Supplementary data

Supplementary Table S1. Biochemical parameters over the time course of 40 study days.

Parameter	d0	d10	d25	d40
**Glucose [mg/dL]**				
HC	110.7 ± 14.5	120.3 ± 20.7	125.2 ± 25.0	113.8 ± 14.5
NC	122.7 ± 25.5	112.3 ± 9.2	106.7 ± 24.5	95.6 ± 9.7
NC+R	103.2 ± 31.5	91.3 ± 6.75	97.0 ± 5.7	100.8 ± 12.8
HC+R	128.3 ± 12.1	113.5 ± 15.1	125.0 ± 17.5	108.3 ± 11.6
**Triglycerides [mg/dL]**				
HC	37.9 ± 13.7	46.6 ± 17.6	51.3 ± 25.6	45.0 ± 17.7
NC	41.8 ± 10.3	50.6 ± 20.3	43.3 ± 13.0	44.4 ± 15.2
NC+R	36.7 ± 14.9	49.0 ± 24.7	26.7 ± 10.6	23.8 ± 10.4
HC+R	35.7 ± 12.4	42.6 ± 20.3	44.5 ± 25.4	27.7 ± 5.9
**Creatinine [mg/dL]**				
HC	1.1 ± 0.1	1.3 ± 0.2	1.4 ± 0.2	1.4 ± 0.3
NC	1.2 ± 0.1	1.4 ± 0.2	1.5 ± 0.2	1.7 ± 0.3
NC+R	1.4 ± 0.1	1.4 ± 0.1	1.6 ± 0.2	1.7 ± 0.1
HC+R	1.2 ± 0.1	1.4 ± 0.2	1.4 ± 0.1	1.4 ± 0.2
**Total protein [g/dL]**				
HC	5.8 ± 0.5	6.6 ± 0.6	7.1 ± 1.0	6.2 ± 0.5
NC	5.7 ± 0.6	6.7 ± 0.7	6.3 ± 0.8	5.9 ± 0.5
NC+R	6.1 ± 0.4	6.5 ± 0.5	6.5 ± 0.5	6.6 ± 0.5
HC+R	5.9 ± 0.3	6.4 ± 0.2	6.8 ± 0.7	7.1 ± 0.8
**GOT [UI/L]**				
HC	28.6 ± 4.1	32.9 ± 3.0	48.4 ± 17.3	51.0 ± 22.6
NC	27.2 ± 5.0	50.4 ± 32.7	20.7 ± 3.8	21.8 ± 3.7
NC+R	29.8 ± 4.5	31.6 ± 7.8	20.5 ± 3.0	19.6 ± 2.9
HC+R	33.7 ± 8.4	34.7 ± 5.2	52.2 ± 17.9	31.4 ± 7.8

All parameters were measured in plasma and serum samples and are within the normal physiological range considering our in-house data for this pig strain at this age. Dara are presented as mean ± SD. d: day. HC: hypercholesterolemic; NC: normocholesterolemic; R: rosuvastatin; GOT: aspartate aminotransferase.

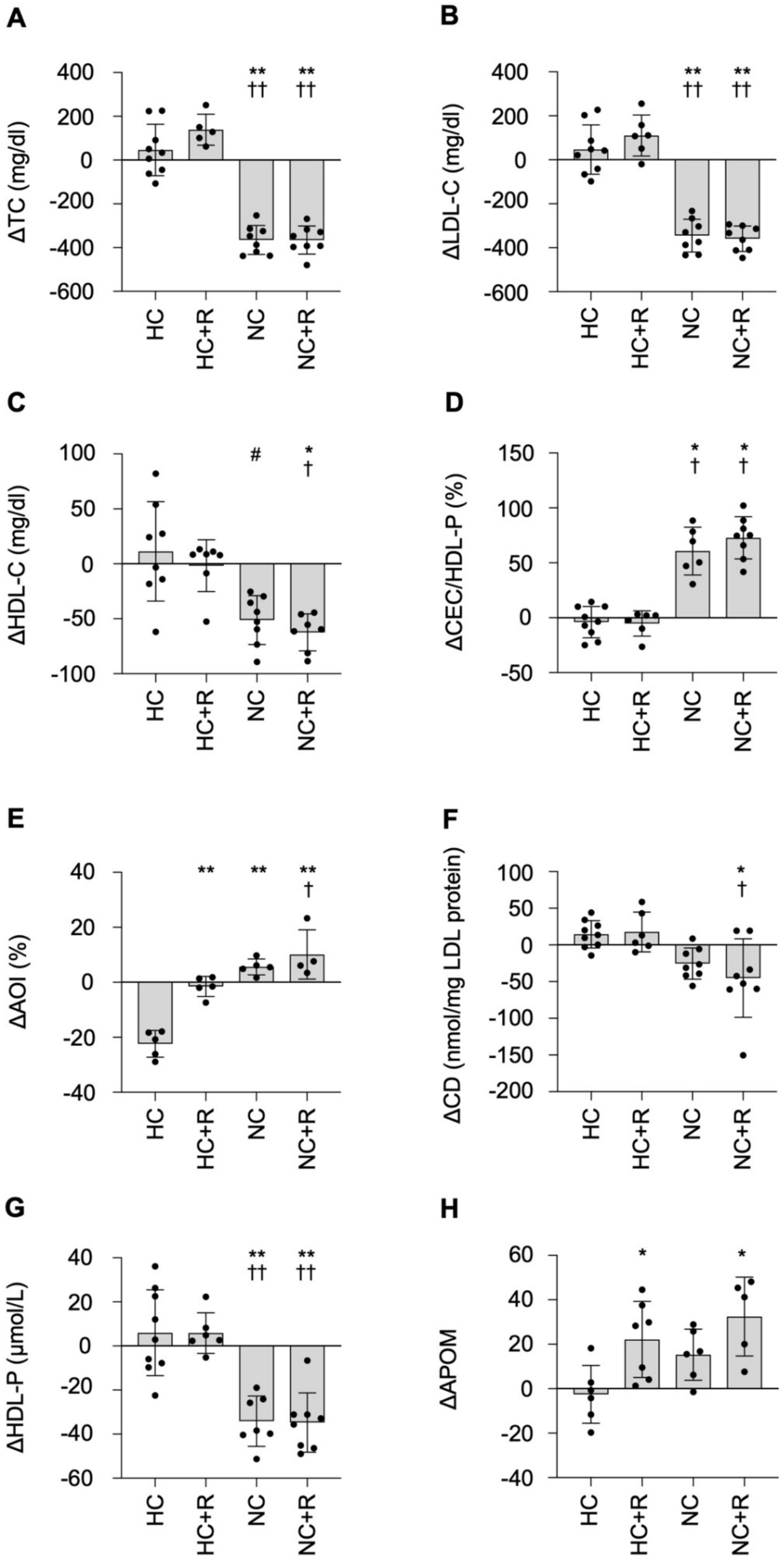
A
ΔTC (mg/dl)
400
200
0
-200
-400
-600
HC
HC+R
NC
NC+R
**
††
B
ΔLDL-C (mg/dl)
C
ΔHDL-C (mg/dl)
100
50
-50
-100
#
*
†
D
ΔCEC/HDL-P (%)
150
E
ΔAOI (%)
40
20
-20
-40
F
ΔCD (nmol/mg LDL protein)
-150
-200
G
ΔHDL-P (μmol/L)
-60
H
ΔAPOM
60

Supplementary Figure S1. The impact of diet and rosuvastatin intervention on all parameters relative to their hypercholesterolemic baseline. The difference (Δ) between day 10 (hypercholesterolemic baseline) and day 40 (end of intervention) of each study group is depicted for TC (**A**), LDL-C (**B**), HDL-C (**C**), CEC/HDL-P (**D**), AOI (**E**), conjugated dienes (**F**), HDL-P (**G**), and HDL-bound APOM levels (**H**).

Delta values are depicted as mean (bars) ± SD (whiskers). Shapiro-Wilk test was applied to test for normality and data analyzed by either nonparametric Kruskal Wallis with Dunn's multicomparison or parametric one-way ANOVA with Tuckey's multicomparison test accordingly. P-values < 0.05 were considered statistically significant. # $p = 0.05$, * $p < 0.05$, ** $p < 0.0001$ in comparison to HC; † $p < 0.05$, †† $p < 0.0001$ in comparison to HC+R.

TC: total cholesterol; LDL-C: low-density lipoprotein; HDL-C: high-density lipoprotein; HDL-P: high-density lipoprotein particle numbers; AOI: antioxidant index; CD: conjugated dienes; CEC: cholesterol efflux capacity; APOM: apolipoprotein M.

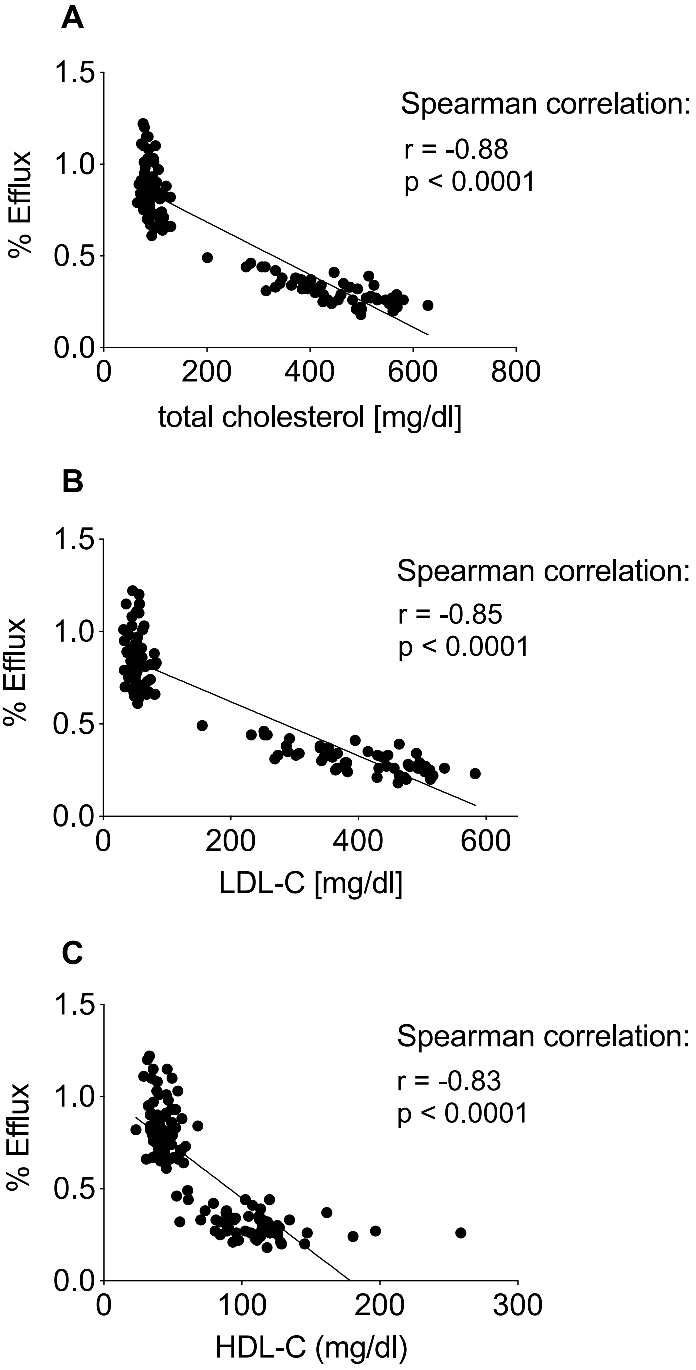

Supplementary Figure S2. Spearman correlations between CEC and lipid parameters. Spearman correlations between CEC and lipid parameters TC, LDL-C and HDL-C are depicted and considered significant with a p-value < 0.05.

TC: total cholesterol; LDL-C: low-density lipoprotein cholesterol; HDL-C: high-density lipoprotein cholesterol.

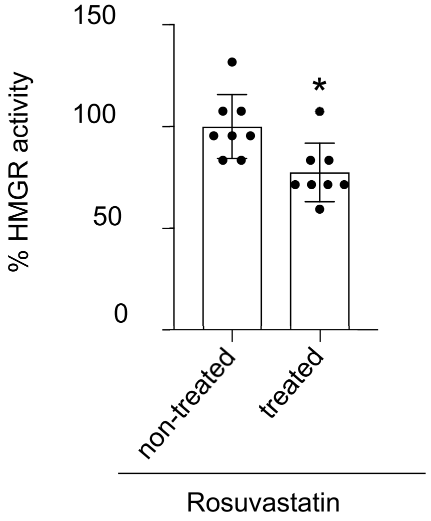

Supplementary Figure S3. Rosuvastatin treatment inhibits hepatic HMG-CoA reductase activity. The effect of rosuvastatin treatment on HMG-CoA reductase activity (%) were measured in representative samples with (n = 8) and without (n = 8) daily rosuvastatin treatment (40mg) at study endpoint. Data is depicted as mean (bars) ± standard deviation (whiskers) on day 40. Shapiro-Wilk test confirmed normality (alpha = 0.05) and data was analyzed by unpaired t-test and considered significant with a p-value < 0.05. * $p < 0.05$ versus non-treated.

HMGR/HMG-CoA reductase: 3-hydroxy-3-methylglutaryl-coenzyme A reductase.
